# RAVAR: a curated repository for rare variant–trait associations

**DOI:** 10.1093/nar/gkad876

**Published:** 2023-10-13

**Authors:** Chen Cao, Mengting Shao, Chunman Zuo, Devin Kwok, Lin Liu, Yuli Ge, Zilong Zhang, Feifei Cui, Mingshuai Chen, Rui Fan, Yijie Ding, Hangjin Jiang, Guishen Wang, Quan Zou

**Affiliations:** Key Laboratory for Bio-Electromagnetic Environment and Advanced Medical Theranostics, School of Biomedical Engineering and Informatics, Nanjing Medical University, Nanjing, China; Key Laboratory for Bio-Electromagnetic Environment and Advanced Medical Theranostics, School of Biomedical Engineering and Informatics, Nanjing Medical University, Nanjing, China; Institute of Artificial Intelligence, Donghua University, Shanghai, China; School of Computer Science, McGill University, Montreal, Canada; Key Laboratory for Bio-Electromagnetic Environment and Advanced Medical Theranostics, School of Biomedical Engineering and Informatics, Nanjing Medical University, Nanjing, China; Key Laboratory for Bio-Electromagnetic Environment and Advanced Medical Theranostics, School of Biomedical Engineering and Informatics, Nanjing Medical University, Nanjing, China; Institute of Fundamental and Frontier Sciences, University of Electronic Science and Technology of China, Chengdu, China; Yangtze Delta Region Institute (Quzhou), University of Electronic Science and Technology of China, Quzhou, China; Institute of Fundamental and Frontier Sciences, University of Electronic Science and Technology of China, Chengdu, China; Yangtze Delta Region Institute (Quzhou), University of Electronic Science and Technology of China, Quzhou, China; Yangtze Delta Region Institute (Quzhou), University of Electronic Science and Technology of China, Quzhou, China; Yangtze Delta Region Institute (Quzhou), University of Electronic Science and Technology of China, Quzhou, China; Yangtze Delta Region Institute (Quzhou), University of Electronic Science and Technology of China, Quzhou, China; Center for Data Science, Zhejiang University, Hangzhou, China; College of Computer Science and Engineering, Changchun University of Technology, Changchun, China; Institute of Fundamental and Frontier Sciences, University of Electronic Science and Technology of China, Chengdu, China; Yangtze Delta Region Institute (Quzhou), University of Electronic Science and Technology of China, Quzhou, China

## Abstract

Rare variants contribute significantly to the genetic causes of complex traits, as they can have much larger effects than common variants and account for much of the missing heritability in genome-wide association studies. The emergence of UK Biobank scale datasets and accurate gene-level rare variant–trait association testing methods have dramatically increased the number of rare variant associations that have been detected. However, no systematic collection of these associations has been carried out to date, especially at the gene level. To address the issue, we present the Rare Variant Association Repository (RAVAR), a comprehensive collection of rare variant associations. RAVAR includes 95 047 high-quality rare variant associations (76186 gene-level and 18 861 variant-level associations) for 4429 reported traits which are manually curated from 245 publications. RAVAR is the first resource to collect and curate published rare variant associations in an interactive web interface with integrated visualization, search, and download features. Detailed gene and SNP information are provided for each association, and users can conveniently search for related studies by exploring the EFO tree structure and interactive Manhattan plots. RAVAR could vastly improve the accessibility of rare variant studies. RAVAR is freely available for all users without login requirement at http://www.ravar.bio.

## Graphical Abstract

**Graphical Abstract:**
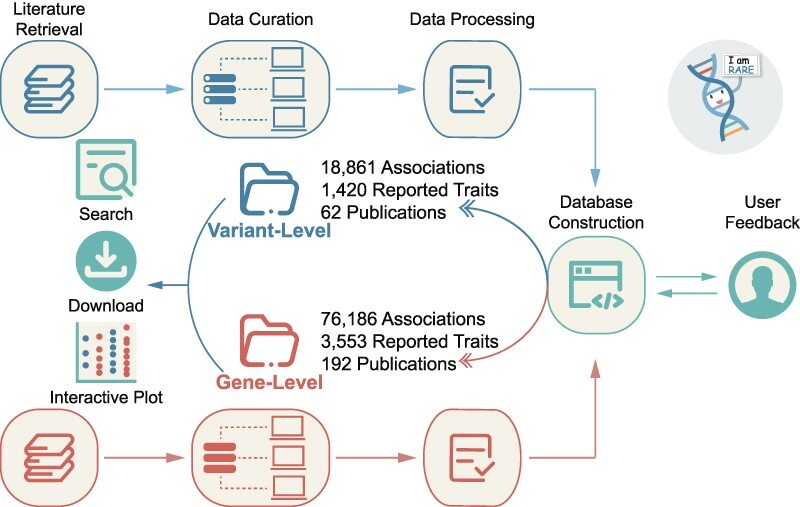

## Introduction

The genome-wide association study (GWAS) has successfully identified thousands of genetic variants associated with complex human traits and diseases (1). However, nearly all variants used in GWAS are common variants that represent merely a fraction of the overall heritability of complex traits and diseases (2). Rare variants play important roles in the genetic causes of complex traits, and could both exert stronger effects and account for a larger proportion of the missing heritability for complex traits/diseases (3–10). Studies have demonstrated that rare variants are associated with numerous traits and diseases such as quantitative lipid traits (11), BMI (12), amyotrophic lateral sclerosis (ALS) (13), schizophrenia and bipolar disorder (14). Analyzing rare variants enables a deeper comprehension of disease-related genetic processes, thereby facilitating the development of personalized therapeutic approaches and early intervention (8).

The widespread application of next-generation sequencing technologies and large cohort studies such as UK Biobank (UKBB) provides an abundance of data for studying rare variant associations. Alongside these large cohort studies, rare variant set testing, particularly with gene-level methods (11,12,14–17), has emerged in recent years to enable researchers to better understand how rare variants may affect genetic mechanisms (11). Compared to single rare variant association tests which lack statistical power in realistic settings, gene-level rare variant testing methods achieve greater power by aggregating multiple rare variants (18).

Although a variety of GWAS resources are currently available, including GWAS Atlas (19), GWAS Catalog (20), GWAS Central (21), CAUSALdb (22), GWASdb (23) and PheGenI (24), none of these existing resources prioritize rare variant associations. Genebass (10) and Brain Catalog (25), focused on UK Biobank data and brain-related GWAS summary statistics respectively. Genebass utilizes single-variant and gene tests on UK Biobank exome-sequence data. Brain Catalog implements variant annotation and gene-based association tests, further enhanced by additional techniques, to unravel brain trait-associated variants, associated genes and functional tissues and cell types. Other gene-based association resources are predominantly focused on curating transcriptome-wide association studies, such as TWAS hub (http://twas-hub.org/), webTWAS (26) and TWAS Atlas (27). Despite the burgeoning volume of rare variant–trait associations and the fact that rare variants exert significantly larger effects than common variants, there remains a lack of resources for curating information on rare variants from publications. The lack of tools for searching and visualizing individual rare variants/genes and their associated various traits hinders the wider recognition and utilization of missing heritability contributed by rare variants.

To surmount these challenges, we introduce RAVAR (RAre VAriant Association Repository), a database meticulously curated with manually selected associations, dedicated to rare variant association studies. RAVAR aggregates a substantial volume of high-quality rare variant–trait associations from a wide array of publications, all of which are annotated with pertinent research metadata. RAVAR utilizes the Experimental Factor Ontology (EFO) (28) mapping and classification framework for traits to standardize nomenclature, definitions, and categorizations. Moreover, RAVAR includes an interactive Manhattan plot for visualizing all identified gene-trait associations and variant–trait associations for a given trait. To date, RAVAR contains 95047 rare variant associations manually curated from 4429 traits across 245 publications, with gene-level associations accounting for 80.5% of the recorded associations. Only 1.6% of associations overlap with GWAS Catalog, which is the most comprehensive GWAS knowledgebase currently available. RAVAR includes state-of-the-art resources and analytical tools relevant to the study of rare variant–trait associations. We expect that RAVAR will significantly bolster the use of rare variant associations for investigating the genetic influences on complex human traits and diseases.

## Materials and methods

### Data curation and standardization

**Figure 1.**
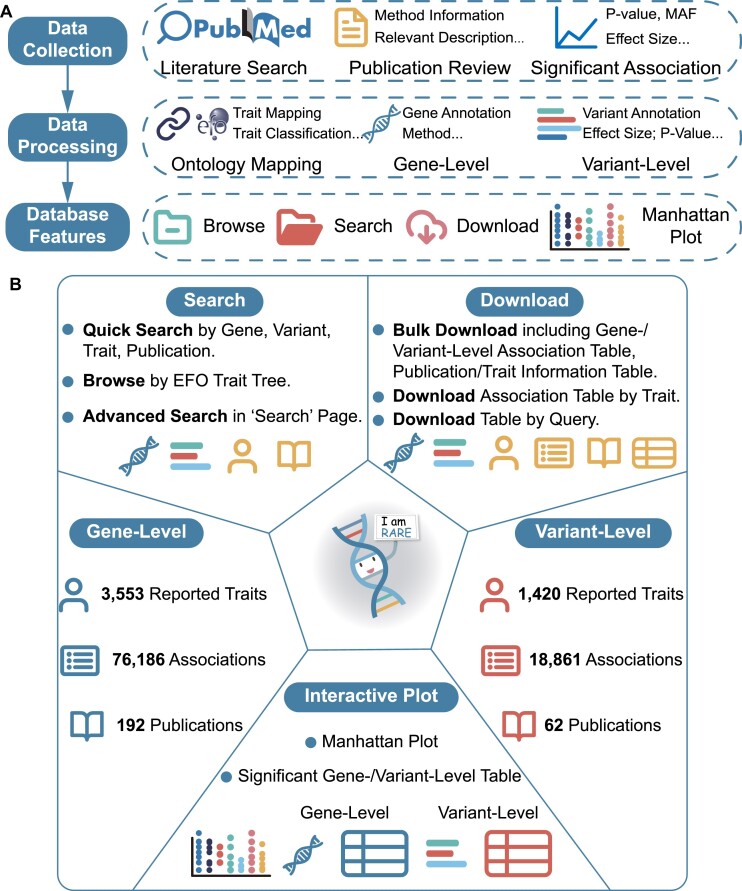
Overview of RAVAR (RAre Variant Association Repository). The figure illustrates the steps by which data is collected, processed, and presented in RAVAR. Also illustrated are the search, download and interactive plotting features of the user interface, along with statistics summarizing the comprehensive gene- and variant-level data in RAVAR.

We conduct a literature search on NCBI PubMed using the predefined keywords ‘rare variant’, and manually select publications with the necessary descriptions (Figure 1A, upper panel). For gene-level associations, RAVAR includes publications which focus on studying human traits using systematic gene-based association testing methods such as the collapsing analysis (8,29,30), sequence kernel association test (SKAT) (15), burden test (14,29,31–33), SAIGE-GENE+ (34), small-sample-adjusted SKAT and the optimal unified test (SKAT-O) (16), adaptive sum of powered score test (aSPU) (35), STAAR-SKAT (11), MetaSTAAR (17), aggregated Cauchy association test (ACAT-V) (36) and several other methods. These methods each uniquely advance rare variant-disease association research. For example, STAAR-SKAT focuses on rare variant associations in non-coding regions, SKAT considers different effect sizes and directions among rare variants, burden tests summarize the cumulative effects of rare variants within a region as a single value, and collapsing analysis groups rare variants within a region into subgroups. In detail, the number of association entries for each method and the corresponding publication are listed in Supplementary Table S1. For each qualified publication, we extract association information including the reported traits, association testing method/software, and P-value indicating statistical significance. For variant-level associations, we additionally record the minor allele frequency (MAF), beta values indicating variant effect sizes, 95% confidence interval, and mapped genes for each variant. Overall, only results with *P*-value less than 1E-4 for gene-level associations or 1E-6 for SNP-level associations are included. Comprehensive information for all qualified publications included in the RAVAR database is listed in Supplementary Table S2.

### Ontology mapping and classification

All genes and variants were reannotated to the Genome Reference Consortium Human Build 38 patch release 14 reference assembly (GRCh38.p14) (Figure 1A, middle panel). In detail, the reannotated information includes the chromosome location, official gene symbol, description, summary, biotype and Ensembl ID of each gene, as well as the chromosome location and genotype of each variant. In order to facilitate user convenience, external links to additional gene and variant details such as Genecards (37), NCBI (38) and Ensembl (39) are provided in the web interface.

To unify the terminology, definitions, and classification of traits, the reported trait/disease information from each publication is standardized by manually mapping terms to the Experimental Factor Ontology (EFO) (28) and establishing well-defined classification criteria. First, we organize and standardize the collected traits by manually deleting publication-specific symbols and ambiguous language. Additionally, we convert any abbreviations to their complete forms to improve the accuracy of mapped terms. It is worth noting that some UKBB phenotypes have been known to cause confusion among researchers due to ambiguities in their naming conventions. We have made extensive efforts to address these issues and ensure accurate trait labeling throughout the database. For example, we describe the trait ‘gamma glutamyltransferase’ in UKBB with the more descriptive term ‘gamma glutamyltransferase measurement’ to improve clarity. In detail, we define ‘originally reported traits’ as traits extracted directly from relevant literature. ‘Reported traits’ refers to traits after trait name preprocessing, and ‘trait label’ and ‘EFO trait label’ are the labels mapped to the EFO. We also reclassify reported traits that are excessively confusing or difficult to interpret under the label ‘Other traits’. Ontology mapping and classification are conducted manually on the corrected labels using the EFO hierarchy tree. To optimize searchability and interoperability, we annotate each trait with crucial information from the original publication including the originally-reported trait, mapped EFO trait label, related publications, ontology ID, description, trait synonyms, and mapped terms. According to the ontological information described above, traits are categorized into the five distinct subcategories of material property, information entity, material entity, process and other traits. These categories enable users to more easily locate and identify traits of interest, thereby facilitating the exploration of the dataset.

### Database implementation

RAVAR runs on Ubuntu Linux (20.04 AMD64) with 32 GB of memory and one 16-core processor. RAVAR is built upon a framework utilizing MySQL and Apache Tomcat Server. The web user interface is developed using a combination of Spring Boot for the back end and HTML5, CSS3, Ajax and Vue.js for the front end. We also use Apache ECharts to generate interactive data visualizations (Figure 1A, bottom panel).

## Results

### Data statistics and ontology mapping results

RAVAR presents a comprehensive collection of 95 047 high-quality rare variant association entries, comprising variant-level and gene-level trait associations. An overview of RAVAR is shown in Figure 1B. The trait screening process identified 245 qualifying publications from a list of 12 261 publications obtained via literature search. These publications cover 12 850 genes in nine categories and 4429 reported human traits in 15 categories, with 4429 reported traits are mapped to 2005 EFO ontology labels. The wide range of genes and traits covered showcases the potential significance of these associations in biological mechanisms studying and clinical applications.

### Single rare variant associations

We define rare variants as single nucleotide polymorphisms (SNPs) with a minor allele frequency (MAF) of <0.02. We then extract a total of 18 861 entries for rare variant-level associations from 62 qualifying publications, corresponding to 6471 distinct variants and 1420 reported traits. Most of the rare variants in our database are associated with more than one mapped trait, with a mean of 1.86 associated traits per SNP. For example, rs2130557010 exhibits associations with 28 mapped traits.

### Gene-level rare variant associations

**Figure 2.**
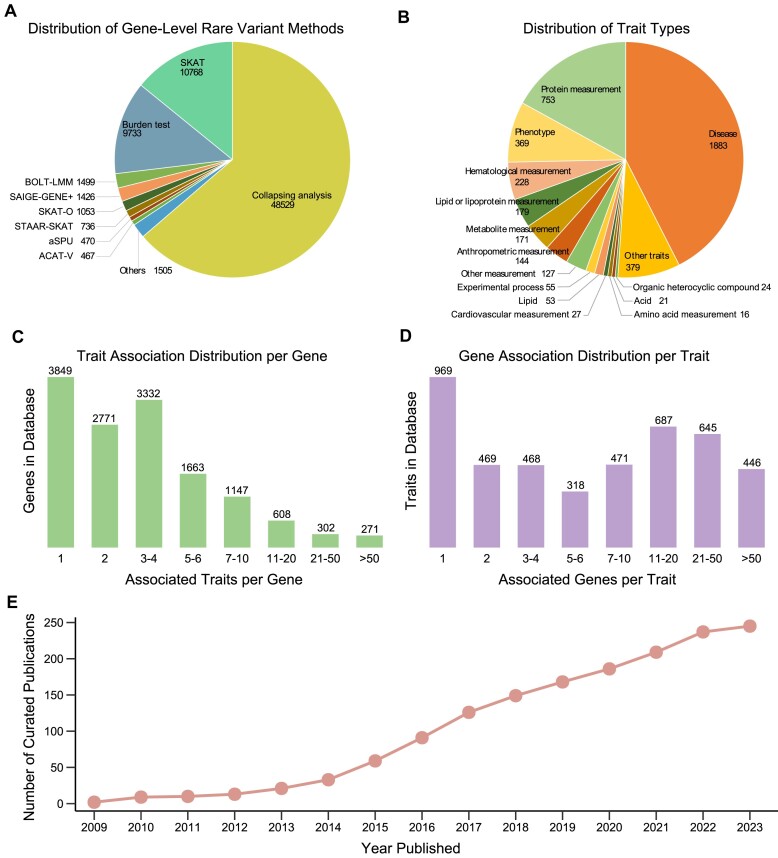
Statistics summarizing the database's contents. (**A**) Distribution of gene-level rare variant associations methods. (**B**) Distribution of traits by type. (**C**) Distribution of genes according to the number of traits each gene is associated with. (**D**) Distribution of traits according to the number of genes each trait is associated with. (**E**) Distribution of curated publications by year published.

We extract a total of 76 186 gene-level rare variant associations from 192 qualifying publications related to 3553 reported traits. These publications employ various gene-based association testing methods/software (Figure 2A), including Collapsing Analysis (48529 associations), SKAT (10768 associations), Burden test (9733 associations), BOLT-LMM (1499 associations), SAIGE-GENE+ (1426 associations), SKAT-O (1053 associations), STAAR-SKAT (736 associations), aSPU (470 associations) and ACAT-V (467 associations). These associations are related to 1468 unique ontology traits (Figure 2B). RAVAR contains 7947 genes associated with at least one trait, of which 7893 are protein-coding genes and 54 are non-coding. On average, each gene is statistically associated with 3.75 different traits (Figure 2C). Meanwhile, most traits in our database exhibit interactions with multiple genes, with a median of seven associated genes per trait (Figure 2D). The annual distribution of the publications included in RAVAR is illustrated in Figure 2E.

### Database usage

The RAVAR website consists of the pages ‘Traits’, ‘Genes’, ‘Variants’ and ‘Publications’, which showcase associations from four perspectives and list related information in interactive tables.

**Figure 3.**
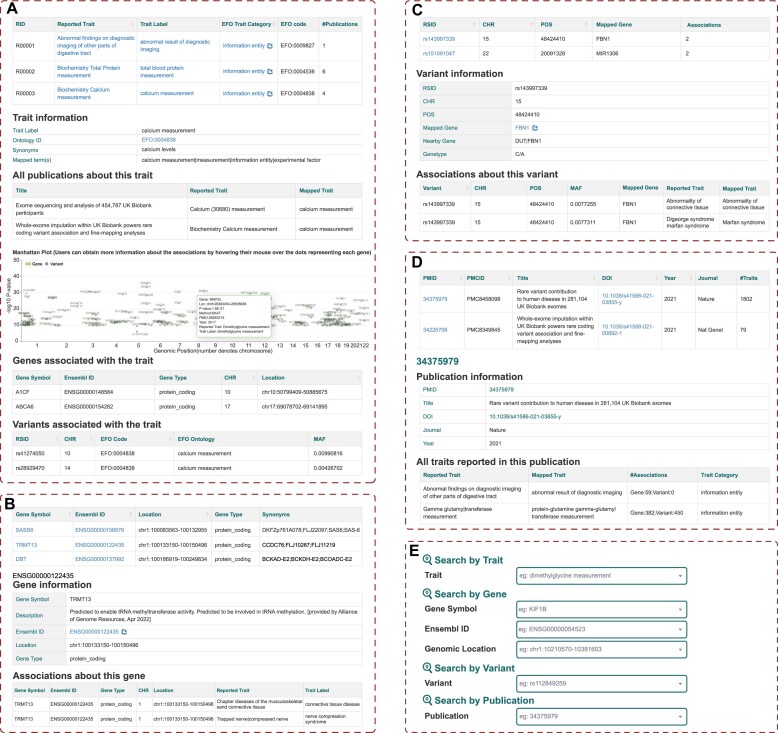
RAVAR functional interfaces. (**A**) Traits interface: Displays EFO trait tree, trait statistics and associated publications. (**B**) Genes interface: Example of the TRMT13 gene. (**C**) Variants interface: Provides details of the rs143997339 variant. (**D**) Publications interface: Example of the publication with PMID 34375979 and its traits. (**E**) Search interface: Offers four channels for traits, genes, variants or publications.

Traits are the core components of the associations reported in RAVAR. On the ‘Traits’ page, the upper left section displays the EFO tree, allowing users to explore categories or traits of interest. This page also includes a summary table with information such as reported traits, EFO ontology trait label, trait category, ontology ID, number of associated publications, and number of association entries. Each trait has its own page containing a more detailed information table for the trait, a table of all related publications, and an interactive Manhattan plot showing gene- or variant-level associations for ease of interpretation (Figure 3A). The Manhattan plot enables users to visualize genomic regions of interest and access detailed information for specific genes/variants. Users can easily access additional information about an association by hovering their mouse over the dots representing each gene/variant within the browser.

Genes and variants are the other key components reported in RAVAR. The pages ‘Genes’ and ‘Variants’ include basic information on gene symbols, gene/variant locations and brief statistical data such as the number of associated entries and the mapped gene for each variant. Each individual gene/variant's page presents more detailed information about that particular gene/variant and its associations. Links to external resources such as dbSNP (40), Ensembl (39), GeneCards (37) and NCBI (38) are provided for additional information retrieval (Figure 3B and C).

On the ‘Publications’ page, detailed information is displayed in an interactive table, including the PMID, title, DOI, publication year, publication journal, and number of associated traits for each publication. Additional information about related traits is displayed on each publication's page (Figure 3D).

Two search methods are provided for efficient querying. First, a search box is provided at the top of the home page to quickly query trait labels, gene symbols, Ensembl IDs, or variant IDs. Additionally, the ‘Search’ page offers an advanced search function from four categories, which means four components of association terms including trait, gene, variant, and publication. This interface allows users to directly search for associations by traits of interest, including trait labels and gene information such as gene symbols, Ensembl IDs, genomic locations, variant IDs, and PMIDs of publications (Figure 3E).

All available data in RAVAR can be downloaded as a excel file for local use. On the ‘Download’ page, all variant/gene-level association data as well as lists summarizing gene information, trait details, and relevant publications are publicly available. Furthermore, users can download association data for specific traits by querying in the search box according to their interests. Users can sort tables in ascending or descending order based on various attributes, providing a more interactive and intuitive experience. RAVAR also offers a ‘Submit’ page for researchers to contribute significant associations that are currently not included in the database. Upon approval by the review committee, these associations, along with detailed information, will be incorporated into the updated version of the database. The ‘Search’ page offers an advanced search function which organizes association entries according to each of the four categories of trait, gene, variant, and publication.

Additionally, a feedback function is available on the ‘Submit’ page, categorized into three types: ‘Issue Report’, ‘Suggestion’ and ‘General’. This enables users to report bugs or issues encountered when using RAVAR, offer suggestions for improvement, and share their thoughts about RAVAR, which will ultimately aid in the continued improvement of the RAVAR database. To acknowledge the receipt of feedback and to further instill confidence in RAVAR among our user community, an automated email notification will be sent to users informing them that the RAVAR team has successfully received their feedback.

## Discussion and future directions

Researchers have been persistently challenged to fully explain the observed heritability of common traits, and considerable efforts have been dedicated to unraveling the causes of this missing heritability. Rare variants, which account for 99% of variations in the human genome, are now recognized as a major contributing factor to the heritability of complex traits and diseases (41). Large-scale cohorts like the Trans-Omics for Precision Medicine (TOPMed) program (5), the Genome Sequencing Program (GSP) and UKBB are currently conducting comprehensive whole genome sequencing/whole exome sequencing studies. Along with the development of gene-based rare variant association tests, these efforts present invaluable opportunities for unraveling the genetic impact of rare variants on the etiology of many complex diseases and traits.

RAVAR presents a substantial number of high-quality, manually curated rare variant associations related to multiple human traits and diseases in an intuitive web interface. RAVAR’s browsing, searching, and visualization features allow users to easily explore rare variant associations and their underlying genetic mechanisms. There are still some limitations in the current version of the RAVAR database. Association entries in this release are manually extracted from literature and lack large-scale systematic analysis. With the emergence of resources like UKBB and TOPMed, along with various rare variant association methods, we will aim to conduct systematic rare association analyses on these extensive GWAS datasets and integrate the results into the database. To ensure ongoing database availability and accuracy, we will establish a regular update and feedback mechanism to reflect the latest findings.

As ongoing effort is essential for maintaining the comprehensiveness and trustworthiness of the RAVAR database, we will continue to integrate more rare variant associations and related information from new publications. Additionally, we intend to enhance RAVAR with an array of comprehensive online tools and data resources in the future to enhance data analysis and interpretation for researchers. Variant-Gene-Trait networks will be implemented to better infer the relationship between rare variants, genes, and traits. In addition, best-practice pipelines for widely recognized rare variant tests will be used to analyze phenotypes derived from the current several large-scale cohorts. Given the rapid increase of publicly available summary-level GWAS data, and the rise of rare variant meta-analysis tools such as MetaSTAAR (17), MetaSKAT (42), RareMetal (43) and SMMAT (44), we will use these developments to identify and catalog rare variant associations from GWAS summary statistics. RAVAR is scheduled to be updated biannually, with the next update expected in January 2024. Taken together, these features will enable RAVAR to become a crucial resource for researchers to enhance our understanding of the role of rare variants in complex human traits and diseases.

## Supplementary Material

gkad876_Supplemental_File

## Data Availability

RAVAR, a meticulously curated repository of rare variant associations, is openly accessible without the requirement for user login. The database can be accessed at http://www.ravar.bio.

## References

[1] Tam V. , Patel N., Turcotte M., Bossé Y., Paré G., Meyre D Benefits and limitations of genome-wide association studies. Nat. Rev. Genet. 2019; 20:467–484. 31068683 10.1038/s41576-019-0127-1

[2] Manolio T.A. , Collins F.S., Cox N.J., Goldstein D.B., Hindorff L.A., Hunter D.J., McCarthy M.I., Ramos E.M., Cardon L.R., Chakravarti A. et al. Finding the missing heritability of complex diseases. Nature. 2009; 461:747–753. 19812666 10.1038/nature08494 PMC2831613

[3] Wainschtein P. , Jain D., Zheng Z., Cupples L.A., Shadyab A.H., McKnight B., Shoemaker B.M., Mitchell B.D., Psaty B.M., Kooperberg C. et al. Assessing the contribution of rare variants to complex trait heritability from whole-genome sequence data. Nat. Genet. 2022; 54:263–273. 35256806 10.1038/s41588-021-00997-7 PMC9119698

[4] Hernandez R.D. , Uricchio L.H., Hartman K., Ye C., Dahl A., Zaitlen N. Ultrarare variants drive substantial cis heritability of human gene expression. Nat. Genet. 2019; 51:1349–1355. 31477931 10.1038/s41588-019-0487-7 PMC6730564

[5] Taliun D. , Harris D.N., Kessler M.D., Carlson J., Szpiech Z.A., Torres R., Taliun S.A.G., Corvelo A., Gogarten S.M., Kang H.M. et al. Sequencing of 53,831 diverse genomes from the NHLBI TOPMed Program. Nature. 2021; 590:290–299. 33568819 10.1038/s41586-021-03205-y PMC7875770

[6] Sun B.B. , Kurki M.I., Foley C.N., Mechakra A., Chen C.Y., Marshall E., Wilk J.B., Chahine M., Chevalier P., Christé G. et al. Genetic associations of protein-coding variants in human disease. Nature. 2022; 603:95–102. 35197637 10.1038/s41586-022-04394-w PMC8891017

[7] Cao C. , Ding B., Li Q., Kwok D., Wu J., Long Q. Power analysis of transcriptome-wide association study: implications for practical protocol choice. PLoS Genet. 2021; 17:e1009405. 33635859 10.1371/journal.pgen.1009405 PMC7946362

[8] Wang Q. , Dhindsa R.S., Carss K., Harper A.R., Nag A., Tachmazidou I., Vitsios D., Deevi S.V.V., Mackay A., Muthas D. et al. Rare variant contribution to human disease in 281,104 UK Biobank exomes. Nature. 2021; 597:527–532. 34375979 10.1038/s41586-021-03855-y PMC8458098

[9] Backman J.D. , Li A.H., Marcketta A., Sun D., Mbatchou J., Kessler M.D., Benner C., Liu D., Locke A.E., Balasubramanian S. et al. Exome sequencing and analysis of 454,787 UK Biobank participants. Nature. 2021; 599:628–634. 34662886 10.1038/s41586-021-04103-z PMC8596853

[10] Karczewski K.J. , Solomonson M., Chao K.R., Goodrich J.K., Tiao G., Lu W., Riley-Gillis B.M., Tsai E.A., Kim H.I., Zheng X. et al. Systematic single-variant and gene-based association testing of thousands of phenotypes in 394,841 UK Biobank exomes. Cell Genom. 2022; 2:100168. 36778668 10.1016/j.xgen.2022.100168 PMC9903662

[11] Li Z. , Li X., Zhou H., Gaynor S.M., Selvaraj M.S., Arapoglou T., Quick C., Liu Y., Chen H., Sun R. et al. A framework for detecting noncoding rare-variant associations of large-scale whole-genome sequencing studies. Nat. Methods. 2022; 19:1599–1611. 36303018 10.1038/s41592-022-01640-x PMC10008172

[12] Jurgens S.J. , Pirruccello J.P., Choi S.H., Morrill V.N., Chaffin M., Lubitz S.A., Lunetta K.L., Ellinor P.T. Adjusting for common variant polygenic scores improves yield in rare variant association analyses. Nat. Genet. 2023; 55:544–548. 36959364 10.1038/s41588-023-01342-w PMC11078202

[13] van Rheenen W. , van der Spek R.A.A., Bakker M.K., van Vugt J., Hop P.J., Zwamborn R.A.J., de Klein N., Westra H.J., Bakker O.B., Deelen P. et al. Common and rare variant association analyses in amyotrophic lateral sclerosis identify 15 risk loci with distinct genetic architectures and neuron-specific biology. Nat. Genet. 2021; 53:1636–1648. 34873335 10.1038/s41588-021-00973-1 PMC8648564

[14] Weiner D.J. , Nadig A., Jagadeesh K.A., Dey K.K., Neale B.M., Robinson E.B., Karczewski K.J., O’Connor L.J Polygenic architecture of rare coding variation across 394,783 exomes. Nature. 2023; 614:492–499. 36755099 10.1038/s41586-022-05684-z PMC10614218

[15] Wu M.C. , Lee S., Cai T., Li Y., Boehnke M., Lin X. Rare-variant association testing for sequencing data with the sequence kernel association test. Am. J. Hum. Genet. 2011; 89:82–93. 21737059 10.1016/j.ajhg.2011.05.029 PMC3135811

[16] Lee S. , Emond M.J., Bamshad M.J., Barnes K.C., Rieder M.J., Nickerson D.A., Christiani D.C., Wurfel M.M., Lin X. Optimal unified approach for rare-variant association testing with application to small-sample case-control whole-exome sequencing studies. Am. J. Hum. Genet. 2012; 91:224–237. 22863193 10.1016/j.ajhg.2012.06.007 PMC3415556

[17] Li X. , Quick C., Zhou H., Gaynor S.M., Liu Y., Chen H., Selvaraj M.S., Sun R., Dey R., Arnett D.K. et al. Powerful, scalable and resource-efficient meta-analysis of rare variant associations in large whole genome sequencing studies. Nat. Genet. 2023; 55:154–164. 36564505 10.1038/s41588-022-01225-6 PMC10084891

[18] Li X. , Li Z., Zhou H., Gaynor S.M., Liu Y., Chen H., Sun R., Dey R., Arnett D.K., Aslibekyan S. et al. Dynamic incorporation of multiple in silico functional annotations empowers rare variant association analysis of large whole-genome sequencing studies at scale. Nat. Genet. 2020; 52:969–983. 32839606 10.1038/s41588-020-0676-4 PMC7483769

[19] Watanabe K. , Stringer S., Frei O., Umićević Mirkov M., de Leeuw C., Polderman T.J.C., van der Sluis S., Andreassen O.A., Neale B.M., Posthuma D A global overview of pleiotropy and genetic architecture in complex traits. Nat. Genet. 2019; 51:1339–1348. 31427789 10.1038/s41588-019-0481-0

[20] Sollis E. , Mosaku A., Abid A., Buniello A., Cerezo M., Gil L., Groza T., Güneş O., Hall P., Hayhurst J. et al. The NHGRI-EBI GWAS Catalog: knowledgebase and deposition resource. Nucleic Acids Res. 2023; 51:D977–D985. 36350656 10.1093/nar/gkac1010 PMC9825413

[21] Beck T. , Rowlands T., Shorter T., Brookes A.J. GWAS Central: an expanding resource for finding and visualising genotype and phenotype data from genome-wide association studies. Nucleic Acids Res. 2023; 51:D986–D993. 36350644 10.1093/nar/gkac1017 PMC9825503

[22] Wang J. , Huang D., Zhou Y., Yao H., Liu H., Zhai S., Wu C., Zheng Z., Zhao K., Wang Z. et al. CAUSALdb: a database for disease/trait causal variants identified using summary statistics of genome-wide association studies. Nucleic Acids Res. 2020; 48:D807–D816. 31691819 10.1093/nar/gkz1026 PMC7145620

[23] Li M.J. , Liu Z., Wang P., Wong M.P., Nelson M.R., Kocher J.P., Yeager M., Sham P.C., Chanock S.J., Xia Z. et al. GWASdb v2: an update database for human genetic variants identified by genome-wide association studies. Nucleic Acids Res. 2016; 44:D869–D876. 26615194 10.1093/nar/gkv1317 PMC4702921

[24] Ramos E.M. , Hoffman D., Junkins H.A., Maglott D., Phan L., Sherry S.T., Feolo M., Hindorff L.A. Phenotype-Genotype Integrator (PheGenI): synthesizing genome-wide association study (GWAS) data with existing genomic resources. Eur. J. Hum. Genet. 2014; 22:144–147. 23695286 10.1038/ejhg.2013.96 PMC3865418

[25] Pan S. , Kang H., Liu X., Lin S., Yuan N., Zhang Z., Bao Y., Jia P. Brain Catalog: a comprehensive resource for the genetic landscape of brain-related traits. Nucleic Acids Res. 2023; 51:D835–D844. 36243988 10.1093/nar/gkac895 PMC9825493

[26] Cao C. , Wang J., Kwok D., Cui F., Zhang Z., Zhao D., Li M.J., Zou Q. webTWAS: a resource for disease candidate susceptibility genes identified by transcriptome-wide association study. Nucleic Acids Res. 2022; 50:D1123–D1130. 34669946 10.1093/nar/gkab957 PMC8728162

[27] Lu M. , Zhang Y., Yang F., Mai J., Gao Q., Xu X., Kang H., Hou L., Shang Y., Qain Q. et al. TWAS Atlas: a curated knowledgebase of transcriptome-wide association studies. Nucleic Acids Res. 2023; 51:D1179–D1187. 36243959 10.1093/nar/gkac821 PMC9825460

[28] Malone J. , Holloway E., Adamusiak T., Kapushesky M., Zheng J., Kolesnikov N., Zhukova A., Brazma A., Parkinson H. Modeling sample variables with an experimental factor ontology. Bioinformatics. 2010; 26:1112–1118. 20200009 10.1093/bioinformatics/btq099 PMC2853691

[29] Li B. , Leal S.M. Methods for detecting associations with rare variants for common diseases: application to analysis of sequence data. Am. J. Hum. Genet. 2008; 83:311–321. 18691683 10.1016/j.ajhg.2008.06.024 PMC2842185

[30] Shugart Y.Y. , Zhu Y., Guo W., Xiong M. Weighted pedigree-based statistics for testing the association of rare variants. Bmc Genomics [Electronic Resource]. 2012; 13:667. 23176082 10.1186/1471-2164-13-667 PMC3827928

[31] Sun J. , Zheng Y., Hsu L. A unified mixed-effects model for rare-variant association in sequencing studies. Genet. Epidemiol. 2013; 37:334–344. 23483651 10.1002/gepi.21717 PMC3740585

[32] Madsen B.E. , Browning S.R. A groupwise association test for rare mutations using a weighted sum statistic. PLoS Genet. 2009; 5:e1000384. 19214210 10.1371/journal.pgen.1000384 PMC2633048

[33] Price A.L. , Kryukov G.V., de Bakker P.I., Purcell S.M., Staples J., Wei L.J., Sunyaev S.R. Pooled association tests for rare variants in exon-resequencing studies. Am. J. Hum. Genet. 2010; 86:832–838. 20471002 10.1016/j.ajhg.2010.04.005 PMC3032073

[34] Zhou W. , Bi W., Zhao Z., Dey K.K., Jagadeesh K.A., Karczewski K.J., Daly M.J., Neale B.M., Lee S. SAIGE-GENE+ improves the efficiency and accuracy of set-based rare variant association tests. Nat. Genet. 2022; 54:1466–1469. 36138231 10.1038/s41588-022-01178-w PMC9534766

[35] Pan W. , Kim J., Zhang Y., Shen X., Wei P. A powerful and adaptive association test for rare variants. Genetics. 2014; 197:1081–1095. 24831820 10.1534/genetics.114.165035 PMC4125385

[36] Liu Y. , Chen S., Li Z., Morrison A.C., Boerwinkle E., Lin X. ACAT: a fast and powerful p value combination method for rare-variant analysis in sequencing studies. Am. J. Hum. Genet. 2019; 104:410–421. 30849328 10.1016/j.ajhg.2019.01.002 PMC6407498

[37] Stelzer G. , Rosen N., Plaschkes I., Zimmerman S., Twik M., Fishilevich S., Stein T.I., Nudel R., Lieder I., Mazor Y. et al. The GeneCards Suite: from gene data mining to disease genome sequence analyses. Curr. Protoc. Bioinformatics. 2016; 54:1.30.31–31.30.33. 10.1002/cpbi.5 27322403

[38] Sayers E.W. , Bolton E.E., Brister J.R., Canese K., Chan J., Comeau D.C., Connor R., Funk K., Kelly C., Kim S. et al. Database resources of the national center for biotechnology information. Nucleic Acids Res. 2022; 50:D20–d26. 34850941 10.1093/nar/gkab1112 PMC8728269

[39] Cunningham F. , Allen J.E., Allen J., Alvarez-Jarreta J., Amode M.R., Armean I.M., Austine-Orimoloye O., Azov A.G., Barnes I., Bennett R. et al. Ensembl 2022. Nucleic Acids Res. 2022; 50:D988–D995. 34791404 10.1093/nar/gkab1049 PMC8728283

[40] Sherry S.T. , Ward M.H., Kholodov M., Baker J., Phan L., Smigielski E.M., Sirotkin K. dbSNP: the NCBI database of genetic variation. Nucleic Acids Res. 2001; 29:308–311. 11125122 10.1093/nar/29.1.308 PMC29783

[41] STAARpipeline: an all-in-one rare-variant tool for biobank-scale whole-genome sequencing data. Nat. Methods. 2022; 19:1532–1533. 36316564 10.1038/s41592-022-01641-w

[42] Lee S. , Teslovich T.M., Boehnke M., Lin X. General framework for meta-analysis of rare variants in sequencing association studies. Am. J. Hum. Genet. 2013; 93:42–53. 23768515 10.1016/j.ajhg.2013.05.010 PMC3710762

[43] Feng S. , Liu D., Zhan X., Wing M.K., Abecasis G.R. RAREMETAL: fast and powerful meta-analysis for rare variants. Bioinformatics. 2014; 30:2828–2829. 24894501 10.1093/bioinformatics/btu367 PMC4173011

[44] Chen H. , Huffman J.E., Brody J.A., Wang C., Lee S., Li Z., Gogarten S.M., Sofer T., Bielak L.F., Bis J.C. et al. Efficient variant set mixed model association tests for continuous and binary traits in large-scale whole-genome sequencing studies. Am. J. Hum. Genet. 2019; 104:260–274. 30639324 10.1016/j.ajhg.2018.12.012 PMC6372261

