# Supplementary material for: RAVAR: a curated repository for rare variant–trait associations
Source: Nucleic Acids Res. 2023 Oct 13;52(D1):D990–7. doi: 10.1093/nar/gkad876 (PMC10767942; doi:10.1093/nar/gkad876)
Supplement: gkad876_Supplemental_File [file gkad876_supplemental_file.pdf]

Supplementary Table S1. Number of association entries for each rare variant association method.

Method/Software	***#Number of association entries***	***PMID of the corresponding publication of Method/Software***
collapsing analysis	48529	23176082; 34375979; 18691683
SKAT	10768	21737059
Burden test	9733	23483651; 36755099; 19214210; 20471002
BOLT-LMM	1499	25642633
SAIGE-GENE+	1426	36138231
SKAT-O	1053	22863193
STAAR-SKAT	736	36303018
aSPU	470	24831820
ACAT-V	467	30849328
F-distributed statistics	163	26058849
LRT	137	27980649
RLRT	131	36088354
TADA model	125	23966865
GATK	108	20644199
F-approximation tests	95	25809955
MetaSTAAR	94	36564505
VEGAS	86	20598278
CMC method	77	18691683
SAIGE-GENE	64	32424355
ExactVCTest	52	27646141
STAR Method	44	32492392
VT	43	20471002
C-alpha	39	21408211
two-stage method	35	19847924
maxT	19	21738570
WSS	18	19214210
AdaJoint	17	24022295
rareMETALS	15	24894501
LF-KM	14	27161037
RQTests	12	28633423
Meta-MultiSKAT	11	31433078
MetaSKAT-O	11	23768515
fitDNM	10	26235986
MetaSKAT	10	23768515
FLM	9	22889854
MAGMA	9	25885710
pVAAST	9	24837662
ARTP method	7	21209154
TASER	5	27152526
MURAT	4	26860061
Cox BT LRT	3	26782979
FB-GGRF	3	27061818
Rasch-based multi-marker association test	3	26379234

ANRV	2	19810025
aSKAT-O	2	22863193
ensemble learning algorithm	2	26619286
GEE	2	24704269
metaFARVAT	2	31275357
uFineMap	2	26458888
ARIEL	1	22441326
GATES	1	21397060
GESE	1	28191685
haplotype method	1	27980634
Hybrid BOMP	1	23358228
Hybrid BOMP+VEST	1	23358228
MiSTF	1	23483651
Positional BOMP	1	23358228
qMSAT	1	22262732
RAML	1	16986161
VT+VEST	1	23358228

Supplementary Table S2. Information for publications collected in the RAVAR database.

PMID	***Title***	***First Author***	***Journal /Book***	***Publication Year***	***DOI***
37301943	Rare genetic variants impact muscle strength	Huang Y	Nat Commun	2023	10.1038/s41467-023-39247-1
37262146	Rare penetrant mutations confer severe risk of common diseases	Fiziev PP	Science	2023	10.1126/science.abo1131
36914870	Schizophrenia risk conferred by rare protein-truncating variants is conserved across diverse human populations	Liu D	Nat Genet	2023	10.1038/s41588-023-01305-1
36809768	Effects of protein-coding variants on blood metabolite measurements and clinical biomarkers in the UK Biobank	Nag A	Am J Hum Genet	2023	10.1016/j.ajhg.2023.02.002
36778668	Systematic single-variant and gene-based association testing of thousands of phenotypes in 394,841 UK Biobank exomes	Karczewski KJ	Cell Genom	2022	10.1016/j.xgen.2022.100168
36672889	Skin Phototype and Disease: A Comprehensive Genetic Approach to Pigmentary Traits Pleiotropy Using PRS in the GCAT Cohort	Farré X	Genes (Basel)	2023	10.3390/genes14010149
36672803	Exome Array Analysis of 9721 Ischemic Stroke Cases from the SiGN Consortium	Xu H	Genes (Basel)	2022	10.3390/genes14010061
36635386	Genomic atlas of the plasma metabolome prioritizes metabolites implicated in human diseases	Chen Y	Nat Genet	2023	10.1038/s41588-022-01270-1
36568030	Whole-exome sequence analysis of anthropometric traits illustrates challenges in identifying effects of rare genetic variants	Young KL	HGG Adv	2022	10.1016/j.xhgg.2022.100163
36564505	Powerful, scalable and resource-efficient meta-analysis of rare variant associations in large whole genome sequencing studies	Li X	Nat Genet	2023	10.1038/s41588-022-01225-6
36450729	Whole-exome sequencing study identifies rare variants and genes associated with intraocular pressure and glaucoma	Gao XR	Nat Commun	2022	10.1038/s41467-022-35188-3
36419110	Whole-exome sequencing identifies novel protein-altering variants associated with serum apolipoprotein and lipid concentrations	Sandholm N	Genome Med	2022	10.1186/s13073-022-01135-6
36333282	The contribution of common and rare genetic variants to variation in metabolic traits in 288,137 East Asians	Kim YJ	Nat Commun	2022	10.1038/s41467-022-34163-2
36311265	Whole-exome sequencing in 415,422 individuals identifies rare variants associated with mitochondrial DNA copy number	Pillalamarri V	HGG Adv	2022	10.1016/j.xhgg.2022.100147
36303018	A framework for detecting noncoding rare-variant associations of large-scale whole-genome sequencing studies	Li Z	Nat Methods	2022	10.1038/s41592-022-01640-x
36297015	Whole Exome Sequencing Study Identifies Novel Rare Risk Variants for Habitual Coffee Consumption Involved in Olfactory Receptor and Hyperphagia	Cheng B	Nutrients	2022	10.3390/nu14204330
36280733	A combined polygenic score of 21,293 rare and 22 common variants improves diabetes diagnosis based on hemoglobin A1C levels	Dornbos P	Nat Genet	2022	10.1038/s41588-022-01200-1
36224396	A saturated map of common genetic variants associated with human height	Yengo L	Nature	2022	10.1038/s41586-022-05275-y

36220816	Whole genome sequence analysis of blood lipid levels in >66,000 individuals	Selvaraj MS	Nat Commun	2022	10.1038/s41467-022-33510-7
36168886	Differences and commonalities in the genetic architecture of protein quantitative trait loci in European and Arab populations	Thareja G	Hum Mol Genet	2023	10.1093/hmg/ddac243
36138231	SAIGE-GENE+ improves the efficiency and accuracy of set-based rare variant association tests	Zhou W	Nat Genet	2022	10.1038/s41588-022-01178-w
36088354	Identifying interpretable gene-biomarker associations with functionally informed kernel-based tests in 190,000 exomes	Monti R	Nat Commun	2022	10.1038/s41467-022-32864-2
36050321	Genetic analyses of the electrocardiographic QT interval and its components identify additional loci and pathways	Young WJ	Nat Commun	2022	10.1038/s41467-022-32821-z
36038634	Large-scale sequencing identifies multiple genes and rare variants associated with Crohn's disease susceptibility	Sazonovs A	Nat Genet	2022	10.1038/s41588-022-01156-2
35982160	Rare coding variation provides insight into the genetic architecture and phenotypic context of autism	Fu JM	Nat Genet	2022	10.1038/s41588-022-01104-0
35954343	Germline Variants Associated with Nasopharyngeal Carcinoma Predisposition Identified through Whole-Exome Sequencing	Lee NY	Cancers (Basel)	2022	10.3390/cancers14153680
35935937	Gene-Based Variant Analysis of Whole-Exome Sequencing in Relation to Eosinophil Count	Höglund J	Front Immunol	2022	10.3389/fimmu.2022.862255
35741860	Identifying Rare Genetic Variants of Immune Mediators as Risk Factors for Autism Spectrum Disorder	Cai C	Genes (Basel)	2022	10.3390/genes13061098
35580180	Impact of natural selection on global patterns of genetic variation and association with clinical phenotypes at genes involved in SARS-CoV-2 infection	Zhang C	Proc Natl Acad Sci U S A	2022	10.1073/pnas.2123000119
35552711	Whole-exome sequencing of 14 389 individuals from the ESP and CHARGE consortia identifies novel rare variation associated with hemostatic factors	Pankratz N	Hum Mol Genet	2022	10.1093/hmg/ddac100
35401413	Deciphering Genetic Susceptibility to Tuberculous Meningitis	Schurz H	Front Neurol	2022	10.3389/fneur.2022.820168
35361970	Polygenic prediction of educational attainment within and between families from genome-wide association analyses in 3 million individuals	Okbay A	Nat Genet	2022	10.1038/s41588-022-01016-z
35177841	Analysis of rare genetic variation underlying cardiometabolic diseases and traits among 200,000 individuals in the UK Biobank	Jurgens SJ	Nat Genet	2022	10.1038/s41588-021-01011-w
35115689	Combined effects of host genetics and diet on human gut microbiota and incident disease in a single population cohort	Qin Y	Nat Genet	2022	10.1038/s41588-021-00991-z
35078996	A genome-wide association study of serum proteins reveals shared loci with common diseases	Gudjonsson A	Nat Commun	2022	10.1038/s41467-021-27850-z
34887591	The power of genetic diversity in genome-wide association studies of lipids	Graham SE	Nature	2021	10.1038/s41586-021-04064-3
34737426	A generalized linear mixed model association tool for biobank-scale data	Jiang L	Nat Genet	2021	10.1038/s41588-021-00954-4
34662886	Exome sequencing and analysis of 454,787 UK Biobank participants	Backman JD	Nature	2021	10.1038/s41586-021-04103-z
34648354	Mapping the proteo-genomic convergence of human diseases	Pietzner M	Science	2021	10.1126/science.abj1541

34626176	Whole-exome sequencing reveals a role of HTRA1 and EGFL8 in brain white matter hyperintensities	Malik R	Brain	2021	10.1093/brain/awab253
34610981	Genetic Studies of Metabolomics Change After a Liquid Meal Illuminate Novel Pathways for Glucose and Lipid Metabolism	Li-Gao R	Diabetes	2021	10.2337/db21-0397
34594039	A cross-population atlas of genetic associations for 220 human phenotypes	Sakaue S	Nat Genet	2021	10.1038/s41588-021-00931-x
34376796	Analyses of biomarker traits in diverse UK biobank participants identify associations missed by European-centric analysis strategies	Sun Q	J Hum Genet	2022	10.1038/s10038-021-00968-0
34375979	Rare variant contribution to human disease in 281,104 UK Biobank exomes	Wang Q	Nature	2021	10.1038/s41586-021-03855-y
34321204	Testosterone and socioeconomic position: Mendelian randomization in 306,248 men and women in UK Biobank	Harrison S	Sci Adv	2021	10.1126/sciadv.abf8257
34260947	Common haplotypes at the CFH locus and low-frequency variants in CFHR2 and CFHR5 associate with systemic FHR concentrations and age-related macular degeneration	Lorés-Motta L	Am J Hum Genet	2021	10.1016/j.ajhg.2021.06.002
34244158	Genetic Analysis of Functional Rare Germline Variants across Nine Cancer Types from an Electronic Health Record Linked Biobank	Shivakumar M	Cancer Epidemiol Biomarkers Prev	2021	10.1158/1055-9965.EPI-21-0082
34230933	Impact of natural selection on global patterns of genetic variation, and association with clinical phenotypes, at genes involved in SARS-CoV-2 infection	Zhang C	medRxiv	2021	10.1101/2021.06.28.21259529
34226706	Whole-exome imputation within UK Biobank powers rare coding variant association and fine-mapping analyses	Barton AR	Nat Genet	2021	10.1038/s41588-021-00892-1
34131117	Genome sequencing unveils a regulatory landscape of platelet reactivity	Keramati AR	Nat Commun	2021	10.1038/s41467-021-23470-9
34125832	Whole-exome sequencing reveals insights into genetic susceptibility to Congenital Zika Syndrome	Borda V	PLoS Negl Trop Dis	2021	10.1371/journal.pntd.0009507
34016428	Identifying susceptibility genes for primary ovarian insufficiency on the high-risk genetic background of a fragile X premutation	Trevino CE	Fertil Steril	2021	10.1016/j.fertnstert.2021.04.021
33919687	Genetic Analysis Reveals Rare Variants in T-Cell Response Gene MR1 Associated with Poor Overall Survival after Urothelial Cancer Diagnosis	Bang L	Cancers (Basel)	2021	10.3390/cancers13081864
33804025	Set-Based Rare Variant Expression Quantitative Trait Loci in Blood and Brain from Alzheimer Disease Study Participants	Patel D	Genes (Basel)	2021	10.3390/genes12030419
33763119	Genome-Wide Identification of Rare and Common Variants Driving Triglyceride Levels in a Nevada Population	Read RW	Front Genet	2021	10.3389/fgene.2021.639418
33679876	Whole-Exome Sequencing and hiPSC Cardiomyocyte Models Identify MYRIP, TRAPPC11, and SLC27A6 of Potential Importance to Left Ventricular Hypertrophy in an African Ancestry Population	Irvin MR	Front Genet	2021	10.3389/fgene.2021.588452
33654115	Identification of candidate genes and pathways in retinopathy of prematurity by whole exome sequencing of preterm infants enriched in phenotypic extremes	Kim SJ	Sci Rep	2021	10.1038/s41598-021-83552-y
33574263	Rare genetic variants affecting urine metabolite levels link population variation to inborn errors of metabolism	Cheng Y	Nat Commun	2021	10.1038/s41467-020-20877-8

33432171	Exome-wide evaluation of rare coding variants using electronic health records identifies new gene-phenotype associations	Park J	Nat Med	2021	10.1038/s41591-020-1133-8
33257650	Identity-by-descent detection across 487,409 British samples reveals fine scale population structure and ultra-rare variant associations	Nait Saada J	Nat Commun	2020	10.1038/s41467-020-19588-x
33230300	Discovery of rare variants associated with blood pressure regulation through meta-analysis of 1.3 million individuals	Surendran P	Nat Genet	2020	10.1038/s41588-020-00713-x
33226994	A phenome-wide association study of 26 mendelian genes reveals phenotypic expressivity of common and rare variants within the general population	Tcheandjieu C	PLoS Genet	2020	10.1371/journal.pgen.1008802
33097823	The genetic architecture of appendicular lean mass characterized by association analysis in the UK Biobank study	Pei YF	Commun Biol	2020	10.1038/s42003-020-01334-0
33093519	Identifying genetic factors that contribute to the increased risk of congenital heart defects in infants with Down syndrome	Trevino CE	Sci Rep	2020	10.1038/s41598-020-74650-4
32929287	Complex genetic signatures in immune cells underlie autoimmunity and inform therapy	Orrù V	Nat Genet	2020	10.1038/s41588-020-0684-4
32888494	The Polygenic and Monogenic Basis of Blood Traits and Diseases	Vuckovic D	Cell	2020	10.1016/j.cell.2020.08.008
32888493	Trans-ethnic and Ancestry-Specific Blood-Cell Genetics in 746,667 Individuals from 5 Global Populations	Chen MH	Cell	2020	10.1016/j.cell.2020.06.045
32492392	Exome Sequencing Identifies Genes and Gene Sets Contributing to Severe Childhood Obesity, Linking PHIP Variants to Repressed POMC Transcription	Marenne G	Cell Metab	2020	10.1016/j.cmet.2020.05.007
32436959	Analysis of putative cis-regulatory elements regulating blood pressure variation	Nandakumar P	Hum Mol Genet	2020	10.1093/hmg/ddaa098
32424355	Scalable generalized linear mixed model for region-based association tests in large biobanks and cohorts	Zhou W	Nat Genet	2020	10.1038/s41588-020-0621-6
32415273	The exhaustive genomic scan approach, with an application to rare-variant association analysis	Kanoungi G	Eur J Hum Genet	2020	10.1038/s41431-020-0639-3
32100372	Convex combination sequence kernel association test for rare-variant studies	Posner DC	Genet Epidemiol	2020	10.1002/gepi.22287
32100327	Risk factors for progression of age-related macular degeneration	Heesterbeek TJ	Ophthalmic Physiol Opt	2020	10.1111/opo.12675
32042192	Using human genetics to understand the disease impacts of testosterone in men and women	Ruth KS	Nat Med	2020	10.1038/s41591-020-0751-5
31992710	Genome-wide rare variant analysis for thousands of phenotypes in over 70,000 exomes from two cohorts	Cirulli ET	Nat Commun	2020	10.1038/s41467-020-14288-y
31866045	UK Biobank Whole-Exome Sequence Binary Phenome Analysis with Robust Region-Based Rare-Variant Test	Zhao Z	Am J Hum Genet	2020	10.1016/j.ajhg.2019.11.012
31748686	An exome-wide rare variant analysis of Korean men identifies three novel genes predisposing to prostate cancer	Oh JJ	Sci Rep	2019	10.1038/s41598-019-53445-2
31433078	Meta-MultiSKAT: Multiple phenotype meta-analysis for region-based association test	Dutta D	Genet Epidemiol	2019	10.1002/gepi.22248
31367044	Exome sequencing of Finnish isolates enhances rare-variant association power	Locke AE	Nature	2019	10.1038/s41586-019-1457-z

31338326	Exome-Wide Rare Variant Analysis From the DiscovEHR Study Identifies Novel Candidate Predisposition Genes for Endometrial Cancer	Shivakumar M	Front Oncol	2019	10.3389/fonc.2019.00574
31275357	metaFARVAT: An Efficient Tool for Meta-Analysis of Family-Based, Case-Control, and Population-Based Rare Variant Association Studies	Wang L	Front Genet	2019	10.3389/fgene.2019.00572
31242253	Pharmacogenomics of statin-related myopathy: Meta-analysis of rare variants from whole-exome sequencing	Floyd JS	PLoS One	2019	10.1371/journal.pone.0218115
31235378	Effectiveness of Intensive Rehabilitation Therapy on Functional Outcomes After Stroke: A Propensity Score Analysis Based on Japan Rehabilitation Database	Kamo T	J Stroke Cerebrovasc Dis	2019	10.1016/j.jstrokecerebrovasdis.2019.06.007
31136621	Exome chip association study excluded the involvement of rare coding variants with large effect sizes in the etiology of anorectal malformations	van de Putte R	PLoS One	2019	10.1371/journal.pone.0217477
31034468	FunSPU: A versatile and adaptive multiple functional annotation-based association test of whole-genome sequencing data	Ma Y	PLoS Genet	2019	10.1371/journal.pgen.1008081
31019915	Novel Complex Interactions between Mitochondrial and Nuclear DNA in Schizophrenia and Bipolar Disorder	Schulmann A	Mol Neuropsychiatry	2019	10.1159/000495658
30976013	Exome array analysis of rare and low frequency variants in amyotrophic lateral sclerosis	Dekker AM	Sci Rep	2019	10.1038/s41598-019-42091-3
30718457	Genetic loci for alcohol-related life events and substance-induced affective symptoms: indexing the "dark side" of addiction	Peng Q	Transl Psychiatry	2019	10.1038/s41398-019-0397-6
30679032	Exome Chip Meta-analysis Fine Maps Causal Variants and Elucidates the Genetic Architecture of Rare Coding Variants in Smoking and Alcohol Use	Brazel DM	Biol Psychiatry	2019	10.1016/j.biopsych.2018.11.024
30665703	Assessing the Pathogenicity, Penetrance, and Expressivity of Putative Disease-Causing Variants in a Population Setting	Wright CF	Am J Hum Genet	2019	10.1016/j.ajhg.2018.12.015
30659681	A large-scale exome array analysis of venous thromboembolism	Lindström S	Genet Epidemiol	2019	10.1002/gepi.22187
30578418	Trans-ethnic association study of blood pressure determinants in over 750,000 individuals	Giri A	Nat Genet	2019	10.1038/s41588-018-0303-9
30382371	Rare variants in the neuronal ceroid lipofuscinosis gene MFSD8 are candidate risk factors for frontotemporal dementia	Geier EG	Acta Neuropathol	2019	10.1007/s00401-018-1925-9
30377230	Genetic Analysis of 400 Patients Refines Understanding and Implicates a New Gene in Atypical Hemolytic Uremic Syndrome	Bu F	J Am Soc Nephrol	2018	10.1681/ASN.2018070759
30315176	Large-scale whole-exome sequencing association studies identify rare functional variants influencing serum urate levels	Tin A	Nat Commun	2018	10.1038/s41467-018-06620-4
30255815	Rare variants in the splicing regulatory elements of EXOC3L4 are associated with brain glucose metabolism in Alzheimer's disease	Miller JE	BMC Med Genomics	2018	10.1186/s12920-018-0390-6
30212457	Exome sequencing-based identification of novel type 2 diabetes risk allele loci in the Qatari population	O'Beirne SL	PLoS One	2018	10.1371/journal.pone.0199837

30206357	Exploring rare and low-frequency variants in the Saguenay-Lac-Saint-Jean population identified genes associated with asthma and allergy traits	Morin A	Eur J Hum Genet	2019	10.1038/s41431-018-0266-4
30175238	Meta-analysis of exome array data identifies six novel genetic loci for lung function	Jackson VE	Wellcome Open Res	2018	10.12688/wellcomeopenres.12583.3
30167848	Rare coding variant analysis in a large cohort of Ashkenazi Jewish families with inflammatory bowel disease	Schiff ER	Hum Genet	2018	10.1007/s00439-018-1927-7
30140000	Deep-coverage whole genome sequences and blood lipids among 16,324 individuals	Natarajan P	Nat Commun	2018	10.1038/s41467-018-05747-8
30108311	Whole exome sequencing study identifies novel rare and common Alzheimer's-Associated variants involved in immune response and transcriptional regulation	Bis JC	Mol Psychiatry	2020	10.1038/s41380-018-0112-7
30012220	Exome-chip meta-analysis identifies novel loci associated with cardiac conduction, including ADAMTS6	Prins BP	Genome Biol	2018	10.1186/s13059-018-1457-6
29898891	Germline Variation and Breast Cancer Incidence: A Gene-Based Association Study and Whole-Genome Prediction of Early-Onset Breast Cancer	Scannell Bryan M	Cancer Epidemiol Biomarkers Prev	2018	10.1158/1055-9965.EPI-17-1185
29875488	Genomic atlas of the human plasma proteome	Sun BB	Nature	2018	10.1038/s41586-018-0175-2
29867916	Evaluating the Genetics of Common Variable Immunodeficiency: Monogenetic Model and Beyond	de Valles-Ibáñez G	Front Immunol	2018	10.3389/fimmu.2018.00636
29852030	Association of Genetic Variants With Response to Anti-Vascular Endothelial Growth Factor Therapy in Age-Related Macular Degeneration	Lorés-Motta L	JAMA Ophthalmol	2018	10.1001/jamaophthalmol.2018.2019
29748316	Common and Rare Coding Genetic Variation Underlying the Electrocardiographic PR Interval	Lin H	Circ Genom Precis Med	2018	10.1161/CIRCGEN.117.002037
29486463	Genetic Variation in Genes Underlying Diverse Dementias May Explain a Small Proportion of Cases in the Alzheimer's Disease Sequencing Project	Blue EE	Dement Geriatr Cogn Disord	2018	10.1159/000485503
29455858	A Large-Scale Multi-ancestry Genome-wide Study Accounting for Smoking Behavior Identifies Multiple Significant Loci for Blood Pressure	Sung YJ	Am J Hum Genet	2018	10.1016/j.ajhg.2018.01.015
29441677	A novel susceptibility locus in MST1 and gene-gene interaction network for Crohn's disease in the Chinese population	Wu WKK	J Cell Mol Med	2018	10.1111/jcmm.13530
29411426	Integrating eQTL data with GWAS summary statistics in pathway-based analysis with application to schizophrenia	Wu C	Genet Epidemiol	2018	10.1002/gepi.22110
29378355	A Genome-wide Study of Common and Rare Genetic Variants Associated with Circulating Thrombin Activatable Fibrinolysis Inhibitor	Stanne TM	Thromb Haemost	2018	10.1160/TH17-04-0249
29321365	Genome-based exome sequencing analysis identifies GYG1, DIS3L and DDRGK1 are associated with myocardial infarction in Koreans	Lee JY	J Genet	2017	10.1007/s12041-017-0854-z

29273807	Protein-altering variants associated with body mass index implicate pathways that control energy intake and expenditure in obesity	Turcot V	Nat Genet	2018	10.1038/s41588-017-0011-x
29263008	Investigating the genetic architecture of dementia with Lewy bodies: a two-stage genome-wide association study	Guerreiro R	Lancet Neurol	2018	10.1016/S1474-4422(17)30400-3
29218897	Codon bias among synonymous rare variants is associated with Alzheimer's disease imaging biomarker	Miller JE	Pac Symp Biocomput	2018	
29190701	A non-threshold region-specific method for detecting rare variants in complex diseases	Hsieh AR	PLoS One	2017	10.1371/journal.pone.0188566
29185836	Exome-chip meta-analysis identifies association between variation in ANKRD26 and platelet aggregation	Chen MH	Platelets	2019	10.1080/09537104.2017.1384538
29155802	Investigation of common, low-frequency and rare genome-wide variation in anorexia nervosa	Huckins LM	Mol Psychiatry	2018	10.1038/mp.2017.88
29093530	Sporadic Hirschsprung Disease: Mutational Spectrum and Novel Candidate Genes Revealed by Next-generation Sequencing	Zhang Z	Sci Rep	2017	10.1038/s41598-017-14835-6
28973304	Exome-wide association study reveals novel psoriasis susceptibility locus at TNFSF15 and rare protective alleles in genes contributing to type I IFN signalling	Dand N	Hum Mol Genet	2017	10.1093/hmg/ddx328
28900119	Analysis of potential protein-modifying variants in 9000 endometriosis patients and 150000 controls of European ancestry	Sapkota Y	Sci Rep	2017	10.1038/s41598-017-10440-9
28879196	Assessment of Whole-Exome Sequence Data in Attempted Suicide within a Bipolar Disorder Cohort	Monson ET	Mol Neuropsychiatry	2017	10.1159/000454773
28867149	Establishing the role of rare coding variants in known Parkinson's disease risk loci	Jansen IE	Neurobiol Aging	2017	10.1016/j.neurobiolaging.2017.07.009
28814775	Mutational profile of rare variants in inflammasome-related genes in Behçet disease: A Next Generation Sequencing approach	Burillo-Sanz S	Sci Rep	2017	10.1038/s41598-017-09164-7
28736464	The Generalized Higher Criticism for Testing SNP-Set Effects in Genetic Association Studies	Barnett I	J Am Stat Assoc	2017	10.1080/01621459.2016.1192039
28642624	Whole Exome Sequencing to Identify Genetic Variants Associated with Raised Atherosclerotic Lesions in Young Persons	Hixson JE	Sci Rep	2017	10.1038/s41598-017-04433-x
28633423	rqt: an R package for gene-level meta-analysis	Zhbannikov IY	Bioinformatics	2017	10.1093/bioinformatics/btx395
28589856	Association analysis of rare variants near the APOE region with CSF and neuroimaging biomarkers of Alzheimer's disease	Nho K	BMC Med Genomics	2017	10.1186/s12920-017-0267-0
28552196	Whole-Genome Sequencing Coupled to Imputation Discovers Genetic Signals for Anthropometric Traits	Tachmazidou I	Am J Hum Genet	2017	10.1016/j.ajhg.2017.04.014

28539126	Knowledge-driven binning approach for rare variant association analysis: application to neuroimaging biomarkers in Alzheimer's disease	Kim D	BMC Med Inform Decis Mak	2017	10.1186/s12911-017-0454-0
28526295	Large-scale exploratory genetic analysis of cognitive impairment in Parkinson's disease	Mata IF	Neurobiol Aging	2017	10.1016/j.neurobiolaging.2017.04.009
28513607	Variants in TTC25 affect autistic trait in patients with autism spectrum disorder and general population	Vojinovic D	Eur J Hum Genet	2017	10.1038/ejhg.2017.82
28480134	Common and rare genetic markers of lipid variation in subjects with type 2 diabetes from the ACCORD clinical trial	Marvel SW	PeerJ	2017	10.7717/peerj.3187
28436151	Whole genome sequence association and ancestry-informed polygenic profile of EEG alpha in a Native American population	Peng Q	Am J Med Genet B Neuropsychiatr Genet	2017	10.1002/ajmg.b.32533
28425186	Association studies of low-frequency coding variants in nonsyndromic cleft lip with or without cleft palate	Leslie EJ	Am J Med Genet A	2017	10.1002/ajmg.a.38210
28417008	Association of gene coding variation and resting metabolic rate in a multi-ethnic sample of children and adults	Hellwege JN	BMC Obes	2017	10.1186/s40608-017-0145-5
28346466	Common and rare exonic MUC5B variants associated with type 2 diabetes in Han Chinese	Chen G	PLoS One	2017	10.1371/journal.pone.0173784
28290481	Identification of Elongated Primary Cilia with Impaired Mechanotransduction in Idiopathic Scoliosis Patients	Oliazadeh N	Sci Rep	2017	10.1038/srep44260
28270201	Exploration of haplotype research consortium imputation for genome-wide association studies in 20,032 Generation Scotland participants	Nagy R	Genome Med	2017	10.1186/s13073-017-0414-4
28265093	Common coding variant in SERPINA1 increases the risk for large artery stroke	Malik R	Proc Natl Acad Sci U S A	2017	10.1073/pnas.1616301114
28263315	Whole-genome sequencing identifies common-to-rare variants associated with human blood metabolites	Long T	Nat Genet	2017	10.1038/ng.3809
28234671	Rare coding variants associated with blood pressure variation in 15 914 individuals of African ancestry	Nandakumar P	J Hypertens	2017	10.1097/HJH.0000000000001319
28199135	Extreme Trait Whole-Genome Sequencing Identifies PTPRO as a Novel Candidate Gene in Emphysema with Severe Airflow Obstruction	Radder JE	Am J Respir Crit Care Med	2017	10.1164/rccm.201606-1147OC
28196273	Sex and Adolescent Ethanol Exposure Influence Pavlovian Conditioned Approach	Madayag AC	Alcohol Clin Exp Res	2017	10.1111/acer.13354
28196272	Deep Sequencing of 71 Candidate Genes to Characterize Variation Associated with Alcohol Dependence	Clark SL	Alcohol Clin Exp Res	2017	10.1111/acer.13352
28195573	Whole-exome sequencing of individuals from an isolated population implicates rare risk variants in bipolar disorder	Lescai F	Transl Psychiatry	2017	10.1038/tp.2017.3
28191685	Gene-based segregation method for identifying rare variants in family-based sequencing studies	Qiao D	Genet Epidemiol	2017	10.1002/gepi.22037

28146470	Rare and low-frequency coding variants alter human adult height	Marouli E	Nature	2017	10.1038/nature21039
28117402	Whole-exome sequencing of 228 patients with sporadic Parkinson's disease	Sandor C	Sci Rep	2017	10.1038/srep41188
28111843	Whole genome sequence study of cannabis dependence in two independent cohorts	Gizer IR	Addict Biol	2018	10.1111/adb.12489
28089252	Practical Approaches for Whole-Genome Sequence Analysis of Heart- and Blood-Related Traits	Morrison AC	Am J Hum Genet	2017	10.1016/j.ajhg.2016.12.009
28061514	A Genome-Wide Association Study of Vertical Cup-Disc Ratio in a Latino Population	Nannini DR	Invest Ophthalmol Vis Sci	2017	10.1167/iovs.16-19891
28011711	Protective coding variants in CFH and PELI3 and a variant near CTRB1 are associated with age-related macular degeneration†	Yu Y	Hum Mol Genet	2016	10.1093/hmg/ddw336
27980654	Joint analysis of multiple blood pressure phenotypes in GAW19 data by using a multivariate rare-variant association test	Sun J	BMC Proc	2016	10.1186/s12919-016-0048-3
27980651	Genetic association analysis based on a joint model of gene expression and blood pressure	Konigorski S	BMC Proc	2016	10.1186/s12919-016-0045-6
27980649	Incorporation of protein binding effects into likelihood ratio test for exome sequencing data	Zhang D	BMC Proc	2016	10.1186/s12919-016-0043-8
27980634	Comparison of multiple single-nucleotide variant association tests in a meta-analysis of Genetic Analysis Workshop 19 family and unrelated data	Wang S	BMC Proc	2016	10.1186/s12919-016-0028-7
27884205	Whole genome sequence analysis of serum amino acid levels	Yu B	Genome Biol	2016	10.1186/s13059-016-1106-x
27863252	The Allelic Landscape of Human Blood Cell Trait Variation and Links to Common Complex Disease	Astle WJ	Cell	2016	10.1016/j.cell.2016.10.042
27764101	Rare Functional Variant in TM2D3 is Associated with Late-Onset Alzheimer's Disease	Jakobsdottir J	PLoS Genet	2016	10.1371/journal.pgen.1006327
27646141	Boosting Gene Mapping Power and Efficiency with Efficient Exact Variance Component Tests of Single Nucleotide Polymorphism Sets	Zhou JJ	Genetics	2016	10.1534/genetics.116.190454
27625572	Whole exome sequencing of extreme age-related macular degeneration phenotypes	Sardell RJ	Mol Vis	2016	
27618447	Trans-ancestry meta-analyses identify rare and common variants associated with blood pressure and hypertension	Surendran P	Nat Genet	2016	10.1038/ng.3654
27559110	Collapsed methylation quantitative trait loci analysis for low frequency and rare variants	Richardson TG	Hum Mol Genet	2016	10.1093/hmg/ddw283
27466198	Analysis with the exome array identifies multiple new independent variants in lipid loci	Kanoni S	Hum Mol Genet	2016	10.1093/hmg/ddw227
27440996	Variation in PTCHD2, CRISP3, NAP1L4, FSCB, and AP3B2 associated with spherical equivalent	Chen F	Mol Vis	2016	
27378695	Exome genotyping arrays to identify rare and low frequency variants associated with epithelial ovarian cancer risk	Permuth JB	Hum Mol Genet	2016	10.1093/hmg/ddw196
27355347	Rare Variants Association Analysis in Large-Scale Sequencing Studies at the Single Locus Level	Jeng XJ	PLoS Comput Biol	2016	10.1371/journal.pcbi.1004993

27346685	Exome Genotyping Identifies Pleiotropic Variants Associated with Red Blood Cell Traits	Chami N	Am J Hum Genet	2016	10.1016/j.ajhg.2016.05.007
27182959	Targeted exonic sequencing of GWAS loci in the high extremes of the plasma lipids distribution	Patel AP	Atherosclerosis	2016	10.1016/j.atherosclerosis.2016.04.011
27161037	Rare-Variant Kernel Machine Test for Longitudinal Data from Population and Family Samples	Yan Q	Hum Hered	2015	10.1159/000445057
27152526	Testing Rare-Variant Association without Calling Genotypes Allows for Systematic Differences in Sequencing between Cases and Controls	Hu YJ	PLoS Genet	2016	10.1371/journal.pgen.1006040
27061818	Testing Allele Transmission of an SNP Set Using a Family-Based Generalized Genetic Random Field Method	Li M	Genet Epidemiol	2016	10.1002/gepi.21970
26964030	Gene and pathway level analyses of germline DNA-repair gene variants and prostate cancer susceptibility using the iCOGS-genotyping array	Saunders EJ	Br J Cancer	2016	10.1038/bjc.2016.50
26782979	Gene-Based Association Analysis for Censored Traits Via Fixed Effect Functional Regressions	Fan R	Genet Epidemiol	2016	10.1002/gepi.21947
26740555	Exome arrays capture polygenic rare variant contributions to schizophrenia	Richards AL	Hum Mol Genet	2016	10.1093/hmg/ddv620
26736064	Exome Sequencing Analysis in Severe, Early-Onset Chronic Obstructive Pulmonary Disease	Qiao D	Am J Respir Crit Care Med	2016	10.1164/rccm.201506-1223OC
26715663	Meta-analysis of Complex Diseases at Gene Level with Generalized Functional Linear Models	Fan R	Genetics	2016	10.1534/genetics.115.180869
26693933	Exome-based case-control association study using extreme phenotype design reveals novel candidates with protective effect in diabetic retinopathy	Shtir C	Hum Genet	2016	10.1007/s00439-015-1624-8
26691988	A large genome-wide association study of age-related macular degeneration highlights contributions of rare and common variants	Fritsche LG	Nat Genet	2016	10.1038/ng.3448
26690388	Exome-wide association analysis reveals novel coding sequence variants associated with lipid traits in Chinese	Tang CS	Nat Commun	2015	10.1038/ncomms10206
26619286	Powerful Tests for Multi-Marker Association Analysis Using Ensemble Learning	Padhukasahasram B	PLoS One	2015	10.1371/journal.pone.0143489
26553438	Recurrent Coding Sequence Variation Explains Only A Small Fraction of the Genetic Architecture of Colorectal Cancer	Timofeeva MN	Sci Rep	2015	10.1038/srep16286
26473621	Identification of Rare Variants in ATP8B4 as a Risk Factor for Systemic Sclerosis by Whole-Exome Sequencing	Gao L	Arthritis Rheumatol	2016	10.1002/art.39449
26458888	Unified tests for fine-scale mapping and identifying sparse high-dimensional sequence associations	Cao S	Bioinformatics	2016	10.1093/bioinformatics/btv586
26394601	Family-based genome scan for age at onset of late-onset Alzheimer's disease in whole exome sequencing data	Saad M	Genes Brain Behav	2015	10.1111/gbb.12250
26379234	A Multi-Marker Genetic Association Test Based on the Rasch Model Applied to Alzheimer's Disease	Wang W	PLoS One	2015	10.1371/journal.pone.0138223
26379185	No Major Host Genetic Risk Factor Contributed to A(H1N1)2009 Influenza Severity	Garcia-Etxebarria K	PLoS One	2015	10.1371/journal.pone.0135983

26366554	Genome sequencing elucidates Sardinian genetic architecture and augments association analyses for lipid and blood inflammatory markers	Sidore C	Nat Genet	2015	10.1038/ng.3368
26264438	Search for new loci and low-frequency variants influencing glioma risk by exome-array analysis	Kinnersley B	Eur J Hum Genet	2016	10.1038/ejhg.2015.170
26252872	Variations in the FRA10AC1 Fragile Site and 15q21 Are Associated with Cerebrospinal Fluid Aβ1-42 Level	Li QS	PLoS One	2015	10.1371/journal.pone.0134000
26235986	Incorporating Functional Information in Tests of Excess De Novo Mutational Load	Jiang Y	Am J Hum Genet	2015	10.1016/j.ajhg.2015.06.013
26229047	Test for rare variants by environment interactions in sequencing association studies	Lin X	Biometrics	2016	10.1111/biom.12368
26199320	Low-frequency germline variants across 6p22.2-6p21.33 are associated with non-obstructive azoospermia in Han Chinese men	Ni B	Hum Mol Genet	2015	10.1093/hmg/ddv257
26105150	Rare and low-frequency variants and their association with plasma levels of fibrinogen, FVII, FVIII, and vWF	Huffman JE	Blood	2015	10.1182/blood-2015-02-624551
26079129	Significant association between rare IPO11-HTR1A variants and attention deficit hyperactivity disorder in Caucasians	Zuo L	Am J Med Genet B Neuropsychiatr Genet	2015	10.1002/ajmg.b.32329
26058849	Gene Level Meta-Analysis of Quantitative Traits by Functional Linear Models	Fan R	Genetics	2015	10.1534/genetics.115.178343
26039597	Mutation burden of rare variants in schizophrenia candidate genes	Girard SL	PLoS One	2015	10.1371/journal.pone.0128988
25963336	Genetic variation in uncontrolled childhood asthma despite ICS treatment	Leusink M	Pharmacogenomics J	2016	10.1038/tpj.2015.36
25961151	Rare and Coding Region Genetic Variants Associated With Risk of Ischemic Stroke: The NHLBI Exome Sequence Project	Auer PL	JAMA Neurol	2015	10.1001/jamaneurol.2015.0582
25958224	The cis and trans effects of the risk variants of coronary artery disease in the Chr9p21 region	Zhao W	BMC Med Genomics	2015	10.1186/s12920-015-0094-0
25809955	Pleiotropy analysis of quantitative traits at gene level by multivariate functional linear models	Wang Y	Genet Epidemiol	2015	10.1002/gepi.21895
25740221	Methods for association analysis and meta-analysis of rare variants in families	Feng S	Genet Epidemiol	2015	10.1002/gepi.21892
25678086	Rare variants at 16p11.2 are associated with common variable immunodeficiency	Maggadottir SM	J Allergy Clin Immunol	2015	10.1016/j.jaci.2014.12.1939
25671699	Pooled sequencing of 531 genes in inflammatory bowel disease identifies an associated rare variant in BTNL2 and implicates other immune related genes	Prescott NJ	PLoS Genet	2015	10.1371/journal.pgen.1004955
25583502	Integrative analysis of sequencing and array genotype data for discovering disease associations with rare mutations	Hu YJ	Proc Natl Acad Sci U S A	2015	10.1073/pnas.1406143112
25519331	Collapsing singletons may boost signal for associating rare variants in sequencing study	Wang W	BMC Proc	2014	10.1186/1753-6561-8-S1-S50

25519320	Rare genetic variant analysis on blood pressure in related samples	Chen H	BMC Proc	2014	10.1186/1753-6561-8-S1-S35
25519312	Identification of rare variants for hypertension with incorporation of linkage information	Chiu YF	BMC Proc	2014	10.1186/1753-6561-8-S1-S109
25516442	Genetic variants associated with longer telomere length are associated with increased lung cancer risk among never-smoking women in Asia: a report from the female lung cancer consortium in Asia	Machiela MJ	Int J Cancer	2015	10.1002/ijc.29393
25488688	Genome-wide association study and admixture mapping reveal new loci associated with total IgE levels in Latinos	Pino-Yanes M	J Allergy Clin Immunol	2015	10.1016/j.jaci.2014.10.033
25360671	Whole exome sequencing identifies novel genes for fetal hemoglobin response to hydroxyurea in children with sickle cell anemia	Sheehan VA	PLoS One	2014	10.1371/journal.pone.0110740
25187575	Association of exome sequences with plasma C-reactive protein levels in >9000 participants	Schick UM	Hum Mol Genet	2015	10.1093/hmg/ddu450
25135348	Genetic variation at glucose and insulin trait loci and response to glucose-insulin-potassium (GIK) therapy: the IMMEDIATE trial	Ellis KL	Pharmacogenomics J	2015	10.1038/tpj.2014.41
25078778	Distribution and medical impact of loss-of-function variants in the Finnish founder population	Lim ET	PLoS Genet	2014	10.1371/journal.pgen.1004494
24876117	Sequencing of LRP2 reveals multiple rare variants associated with urinary trefoil factor-3	McMahon GM	J Am Soc Nephrol	2014	10.1681/ASN.2013111240
24728188	No large-effect low-frequency coding variation found for myocardial infarction	Holmen OL	Hum Mol Genet	2014	10.1093/hmg/ddu175
24561134	Exome sequencing implicates an increased burden of rare potassium channel variants in the risk of drug-induced long QT interval syndrome	Weeke P	J Am Coll Cardiol	2014	10.1016/j.jacc.2014.01.031
24507775	Whole-exome sequencing identifies rare and low-frequency coding variants associated with LDL cholesterol	Lange LA	Am J Hum Genet	2014	10.1016/j.ajhg.2014.01.010
24507774	Association of low-frequency and rare coding-sequence variants with blood lipids and coronary heart disease in 56,000 whites and blacks	Peloso GM	Am J Hum Genet	2014	10.1016/j.ajhg.2014.01.009
24212883	A large-scale screen for coding variants predisposing to psoriasis	Tang H	Nat Genet	2014	10.1038/ng.2827
24159917	Gastrointestinal stromal tumors: a case-only analysis of single nucleotide polymorphisms and somatic mutations	O'Brien KM	Clin Sarcoma Res	2013	10.1186/2045-3329-3-12
24151499	New insights into the genetic mechanism of IQ in autism spectrum disorders	Wang HZ	Front Genet	2013	10.3389/fgene.2013.00195
24022295	A fast multilocus test with adaptive SNP selection for large-scale genetic-association studies	Zhang H	Eur J Hum Genet	2014	10.1038/ejhg.2013.201
23966874	A flexible approach for the analysis of rare variants allowing for a mixture of effects on binary or quantitative traits	Clarke GM	PLoS Genet	2013	10.1371/journal.pgen.1003694
23891470	Meta-analysis of gene-level associations for rare variants based on single-variant statistics	Hu YJ	Am J Hum Genet	2013	10.1016/j.ajhg.2013.06.011

23778322	Rare SERINC2 variants are specific for alcohol dependence in individuals of European descent	Zuo L	Pharmacogenet Genomics	2013	10.1097/FPC.0b013e328362f9f2
23483651	A unified mixed-effects model for rare-variant association in sequencing studies	Sun J	Genet Epidemiol	2013	10.1002/gepi.21717
23358228	A hybrid likelihood model for sequence-based disease association studies	Chen YC	PLoS Genet	2013	10.1371/journal.pgen.1003224
23263489	Exome array analysis identifies new loci and low-frequency variants influencing insulin processing and secretion	Huyghe JR	Nat Genet	2013	10.1038/ng.2507
23176082	Weighted pedigree-based statistics for testing the association of rare variants	Shugart YY	BMC Genomics	2012	10.1186/1471-2164-13-667
22889854	Quantitative trait locus analysis for next-generation sequencing with the functional linear models	Luo L	J Med Genet	2012	10.1136/jmedgenet-2012-100798
22641211	Exome sequencing and the genetic basis of complex traits	Kiezun A	Nat Genet	2012	10.1038/ng.2303
21738570	From SNPs to genes: disease association at the gene level	Lehne B	PLoS One	2011	10.1371/journal.pone.0020133
20876667	Genome-wide significant associations for variants with minor allele frequency of 5% or less--an overview: A HuGE review	Panagiotou OA	Am J Epidemiol	2010	10.1093/aje/kwq234
20852893	Rare variation at the TNFAIP3 locus and susceptibility to rheumatoid arthritis	Bowes J	Hum Genet	2010	10.1007/s00439-010-0889-1
20691247	A genome-wide association study of Cloninger's temperament scales: implications for the evolutionary genetics of personality	Verweij KJ	Biol Psychol	2010	10.1016/j.biopsycho.2010.07.018
20549515	Genome-wide searching of rare genetic variants in WTCCC data	Feng T	Hum Genet	2010	10.1007/s00439-010-0849-9
20433910	Genome-wide approaches to schizophrenia	Duan J	Brain Res Bull	2010	10.1016/j.brainresbull.2010.04.009
20421590	Genetic causes of high and low serum HDL-cholesterol	Weissglas-Volkov D	J Lipid Res	2010	10.1194/jlr.R004739
19847924	Detecting rare variants for complex traits using family and unrelated data	Zhu X	Genet Epidemiol	2010	10.1002/gepi.20449
19571035	Finding common susceptibility variants for complex disease: past, present and future	Panoutsopoulou K	Brief Funct Genomic Proteomic	2009	10.1093/bfgp/elp020
19264985	Rare variants of IFIH1, a gene implicated in antiviral responses, protect against type 1 diabetes	Nejentsev S	Science	2009	10.1126/science.1167728
